# Traumatic Thoracic Aortic Injury in a Three-Year-Old Patient: A Case Report

**DOI:** 10.7759/cureus.33117

**Published:** 2022-12-30

**Authors:** Tarfah AlSayyari, Zahra Almatar, Abdulaziz AlShomar, Mohammad Alnamshan

**Affiliations:** 1 College of Medicine/Surgery, King Saud Bin Abdulaziz University for Health Sciences, Riyadh, SAU; 2 Pediatric Surgery, King Faisal Specialist Hospital and Research Centre, Riyadh, SAU; 3 Vascular Surgery, King Abdulaziz Medical City, National Guard, Riyadh, SAU; 4 Pediatric Surgery, King Abdulaziz Medical City, National Guard, Riyadh, SAU

**Keywords:** traumatic aortic injury, blunt thoracic trauma, pediatric, aortic trauma, aortic injury

## Abstract

Management of a traumatic ruptured aorta in the pediatric population is quite challenging. Options vary, with each having its own morbidity, and include open repair, endovascular stent grafts, and/or anti-impulse therapy. Although endovascular stenting is an emerging management modality in traumatic aortic injury in adults, open repair is still the gold standard in the pediatric population.

In this case, we reported the survival of a three-year-old boy who underwent successful surgical repair with a Dacron graft and anastomosis after an acute traumatic thoracic aortic pseudoaneurysm with mediastinal hematoma.

## Introduction

Traumatic aortic injury is one of the most common causes of death in pediatric blunt trauma, with an incidence rate of between 0.05% to 7.4% in children with major chest trauma, depending on the population [1,2]. Most of the cases present after a motor vehicle accident (MVA), and not wearing seatbelts is a major risk factor [3,4].

Management options for traumatic rupture of the aorta (TRA) have evolved to include nonoperative anti-impulse therapy, endovascular stent graft, and open repair with or without a synthetic graft. Open repair has been the gold standard for traumatic aortic injuries in the pediatric population in most centers [3,5].

## Case presentation

A three-year-old boy with acute lymphocytic leukemia on chemotherapy protocol was brought to the emergency department with steering wheel trauma after being involved in a motor vehicle accident while he was unrestrained and sitting on his father’s lap.

The primary survey showed an intact airway, with equal bilateral air entry, no external bleeding, and normal vital signs with a blood pressure of 109/52mmHg and a pulse rate of 116 pulses per minute. The Glasgow Coma Scale (GCS) was 15/15. The physical examination revealed a small abrasion involving the face and upper chest wall with no other external findings. Chest and pelvis X-rays were normal.

**Figure 1:**
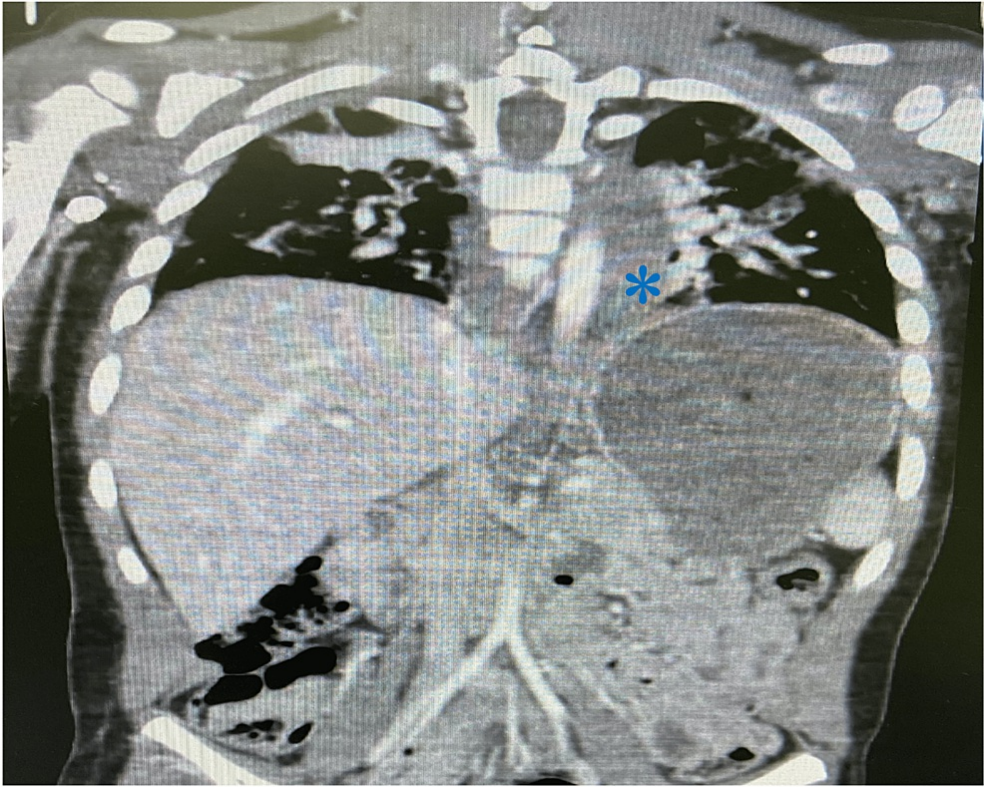
CT chest showing grade III aortic pseudoaneurysm (asterisk )

**Figure 2:**
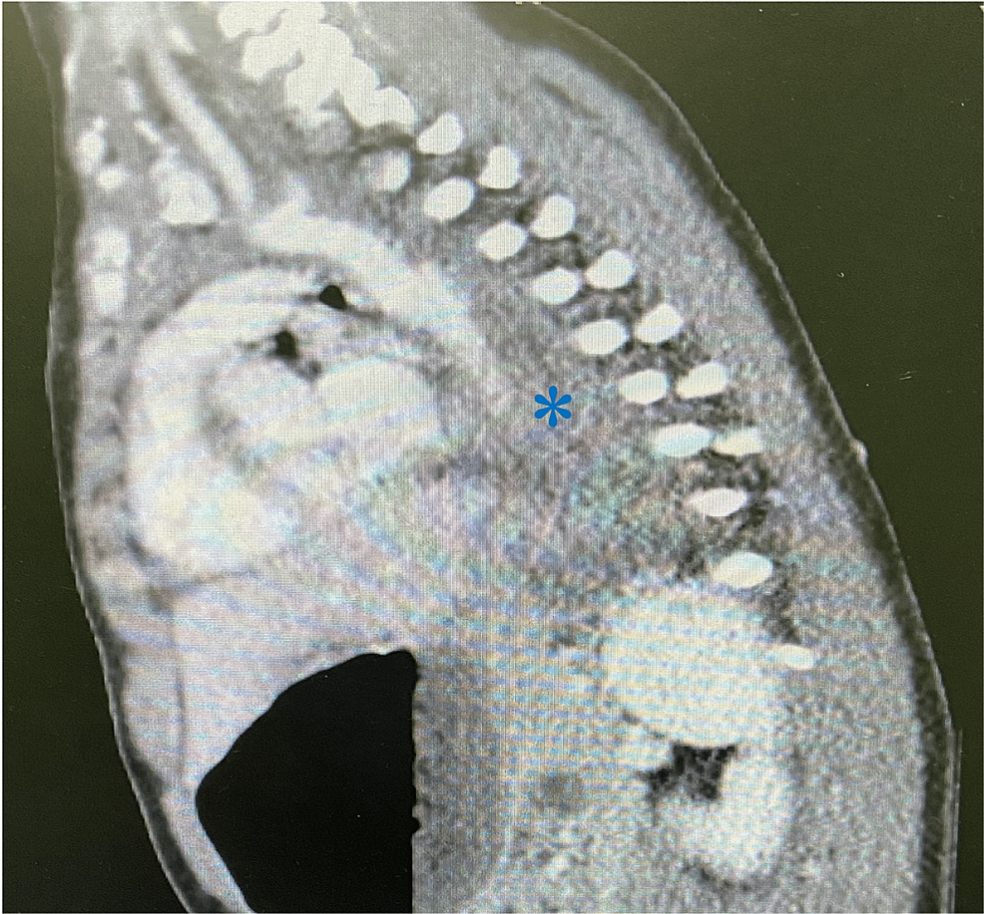
CT sagittal view showing grade III aortic pseudoaneurysm (asterisk)

Pan computed tomography (CT) was done as part of the advanced trauma life support protocol and showed moderate brain edema, an avulsion fracture of the C1 left facet, a right sixth rib fracture, bilateral lung contusion, and acute traumatic thoracic aortic pseudoaneurysm with mediastinal hematoma (Figures 1, 2), as well as a left renal laceration Grade 1.

A multidisciplinary approach, involving pediatric, cardiothoracic, and vascular surgery, along with interventional radiology services, was employed to manage the case. The parents were informed about the risks and benefits associated with all options. The decision was made to proceed with the open aortic repair, with a high-risk death on table consent obtained. 

**Figure 3:**
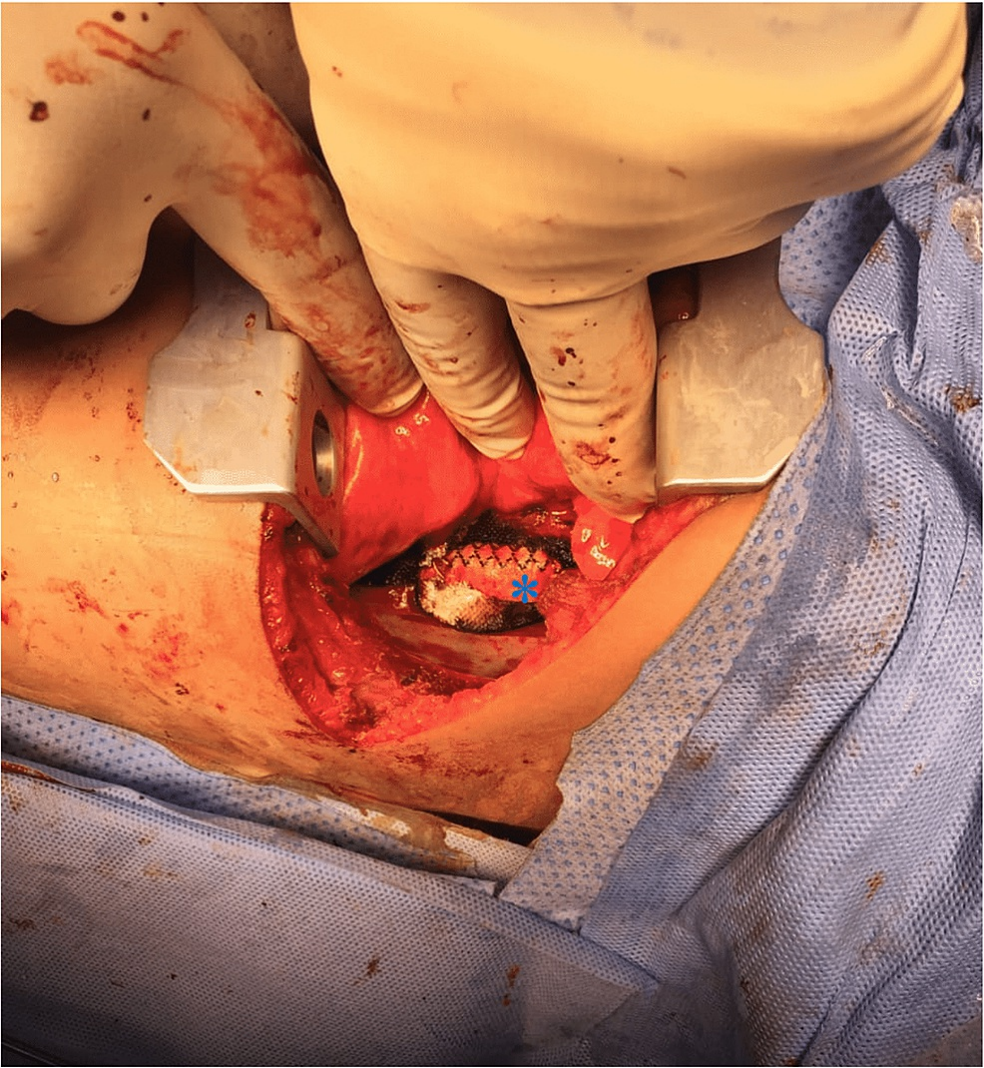
Intraoperative picture of the aortic pseudoaneurysm after excision of the disrupted segment and application of the Dacron graft (asterisk)

The patient underwent a left posterolateral thoracotomy. Intraoperatively, there was a left congested lung with hemothorax and periaortic hematoma and a 1.5 cm disruption at the mid-descending thoracic aorta. An excision of the disrupted segment was made and a 2 cm length Dacron graft was applied. Proximal and distal were anastomosed and the total time of the aortic cross-clamp was approximately 30-35 minutes (Figure 3).

The patient was given a heparin infusion immediately after the operation, along with antibiotics and milrinone. On day one, postoperatively, femoral pulses were weak in the left leg and absent in the right. Therefore, the patient underwent a femoral embolectomy, and pulses were gained on the same day. On the second day, pulses were intact in both legs, but the patient was not able to move the lower limbs. Five days after, the patient developed deep venous thrombosis in the right external iliac and femoral veins and was started on therapeutic anticoagulants. In the third week, the patient was extubated and discharged, after he completed his chemotherapy sessions and was referred for rehabilitation service.

 He was followed up for a period of four years. The lower limbs remained partially paralyzed, and he was admitted recently for scoliosis correction.

## Discussion

Traumatic aortic injuries are classified into four grades based on the CT radiological findings: grade I intimal tear, grade II intramural hematoma, grade III traumatic pseudoaneurysm, and grade IV rupture of the aorta [6].

Management options for TRA range from open repair, to the use of endovascular stent-grafts, and lastly, the use of anti-impulse therapy, which includes the use of beta blockers and control of blood pressure [2,3]. The decision on the management is individualized based on many factors, including the patient’s stability, the grade of the injury, other associated injuries, available options in the institute, and how skilled the surgeon is.

A series by Riyadh Karmy-Jones reported a traumatic rupture of the thoracic aorta in 11 pediatric patients. Of these, six underwent operative repair, including a two-year-old toddler. Their survival rate was nearly 100% with complete recovery for all patients except for one case where the patient was operated on with no bypass and survived with partial lower-limb paralysis. On the other hand, an endovascular repair was done on three patients, of which two died due to head injuries, and only one survived. Two patients were managed non-operatively, of which one survived, while the other one died due to a massive head injury [7].

It is well-documented in studies and metanalysis that the use of cardiac bypass during aortic repair decreases the risk of spinal cord ischemia and subsequent paralysis. Incidences of paraplegia were found to be lower among those who had been operated on using full or partial bypass compared to using simple cross clamp along with a percentage of 5.2% and 25.5%, respectively [8]. Applying bypass, on the other hand, requires more operative time and needs full heparinization, which should be used with caution in patients with multiple trauma and might be challenging in small children with difficult cannulation and that was the case in our patient [9]. Another study that reviewed a clinical course of three pediatric patients aged four, 10, and 16 years showed a successful operative repair with the use of partial left heart bypass during aortic cross-clamping in all three. Aortic reconstruction techniques used in those patients included primary suture repair in the four-year-old patient, interposition conduit in the 10-year-old one, and patch angioplasty in the older patient [10]. A retrospective review by Scott A. Anderson of all patients admitted with blunt aortic injuries for a period of 10 years from birth to 19 years old demonstrates 11 patients with aortic injuries. Those with minor injuries were managed nonoperatively with no complications, while four patients with thoracic aortic damage were managed with open repair with complications, including paraplegia, renal failure, recurrent laryngeal nerve injury, and pulmonary embolism [11]. These are well-known complications in cases where an aortic injury is managed by open repair, especially if the cardiopulmonary bypass was not utilized.

Although open vascular repair has been the gold standard for aortic traumatic injuries in children, thoracic endovascular aortic repair (TEVAR) has recently gained popularity in the management of pediatric cases as it is the preferred method in the adult population [12-14]. TEVAR has been associated with a lower incidence of morbidity and mortality in adult literature. This modality has been used successfully in several cases of adolescent thoracic pediatric trauma, especially in subacute or chronic aortic pseudoaneurysms [15-17]. In one case, medical management was used as a bridge until the appropriate stent was available in a stable 11-year-old with a thoracic pseudoaneurysm, as reported by Gustavo Stringel [18]. Zenith Alpha reviewed 16 previous cases who underwent endovascular repair with ages ranging from 11 to 17 years and showed a mortality rate of 16 % due to non-aortic associated injuries [13].

The endovascular repair can be an alternative option in emergent settings when there are associated injuries that could complicate the open repair [19]. However, it wasn’t an option for our patient mainly due to the small diameter of the aorta and access vessels at his age, which may not accommodate the size of the delivery device [17]. The lack of long-term follow-up data and the possibility of perforation, leak, migration, and pseudo-coarctation were also reasons for not preferring this approach [19].

Conservative management as a solo option has a high mortality rate reaching up to 46% in Grade III aortic injuries. Medical management should be used with caution in patients with significant brain injuries, multiple trauma, and low systolic blood pressure [12,13,18]. Although there have been some reported cases of good short outcomes with conservative management, it is commonly used as a bridge until the patient is stable enough for a definite repair [20].

## Conclusions

In conclusion, the diagnosis of contained thoracic injury in the pediatric population requires a high index of suspicion. We advocate for a low threshold for chest imaging in any child with the suspected mechanism. Open repair is still the gold standard for such cases. In order to decrease the complications associated with operative management in cases where it’s not feasible to use an endovascular stent, we recommend applying the repair while using a cardiopulmonary bypass whenever it is possible.
